# The *trans* fatty acid elaidate affects the global DNA methylation profile of cultured cells and in vivo

**DOI:** 10.1186/s12944-016-0243-2

**Published:** 2016-04-12

**Authors:** José Flores-Sierra, Martín Arredondo-Guerrero, Braulio Cervantes-Paz, Dalia Rodríguez-Ríos, Yolanda Alvarado-Caudillo, Finn C. Nielsen, Katarzyna Wrobel, Kazimierz Wrobel, Silvio Zaina, Gertrud Lund

**Affiliations:** Department of Medical Sciences, Division of Health Sciences, Leon Campus, University of Guanajuato, Leon, Gto. Mexico; Department of Chemistry, Division of Natural and Exact Sciences, Guanajuato Campus, University of Guanajuato, Guanajuato, Gto. Mexico; Tecnológico de Monterrey, Leon Campus, Leon, Gto. Mexico; Department of Genetic Engineering, CINVESTAV Irapuato Unit, 36821 Irapuato, Gto. Mexico; Center for Genomic Medicine, Rigshospitalet, University of Copenhagen, Copenhagen, Denmark

**Keywords:** DNA methylation, Whole genome expression, Elaidic acid, Oleic acid, THP-1 cell line, Mouse model

## Abstract

**Background:**

The deleterious effects of dietary *trans* fatty acids (tFAs) on human health are well documented. Although significantly reduced or banned in various countries, tFAs may trigger long-term responses that would represent a valid human health concern, particularly if tFAs alter the epigenome.

**Methods:**

Based on these considerations, we asked whether the tFA elaidic acid (EA; tC18:1) has any effects on global DNA methylation and the transcriptome in cultured human THP-1 monocytes, and whether the progeny of EA-supplemented dams during either pregnancy or lactation in mice (*n* = 20 per group) show any epigenetic change after exposure.

**Results:**

EA induced a biphasic effect on global DNA methylation in THP-1 cells, i.e. hypermethylation in the 1–50 μM concentration range, followed by hypomethylation up to the 200 μM dose. On the other hand, the *cis* isomer oleic acid (OA), a fatty acid with documented beneficial effects on human health, exerted a distinct response, i.e. its effects were weaker and only partially overlapping with EA’s. The maximal differential response between EA and OA was observed at the 50 μM dose. Array expression data revealed that EA induced a pro-inflammatory and adipogenic transcriptional profile compared with OA, although with modest effects on selected (*n* = 9) gene promoter methylation. In mice, maternal EA supplementation in utero or via the breastmilk induced global adipose tissue DNA hypermethylation in the progeny, that was detectable postnatally at the age of 3 months.

**Conclusion:**

We document that global DNA hypermethylation is a specific and consistent response to EA in cell culture and in mice, and that EA may exert long-term effects on the epigenome following maternal exposure.

**Electronic supplementary material:**

The online version of this article (doi:10.1186/s12944-016-0243-2) contains supplementary material, which is available to authorized users.

## Background

Fatty acid (FA) *trans* isomers (tFAs) produced by fat hydrogenation in the food processing industry have been extensively linked to pathologies such as cardiovascular disease, diabetes and obesity [1]. The pathogenic effects of tFAs have been attributed to biochemical alterations in cholesterol metabolism and structural changes in biomembranes, i.e. an increase in membrane rigidity due to the disruption of the ordered structure of the lipid bilayer [2]. As a result, the legislation of several countries bans or limits the content of tFA in processed food, leading to a perceived lesser relevance for the topic of tFAs in human health (see: www.tfx.org.uk/page116.html for one of the earliest examples of tFA-banning laws). Yet, FA-rich lipoproteins and individual FAs including arachidonic, oleic and palmitic acid (AA, OA and PA, respectively) can modify the DNA methylome [3–5] (Silva-Martínez et al., in press), adding to a large number of other substances identified by nutritional epigenetics over the last decade [6, 7]. This body of evidence raises the question whether tFAs can modify the epigenome and therefore may exert long-term or transgenerational effects. To our knowledge, the effects of tFAs on DNA methylation have not been studied, besides the intriguing observation that the activity of the DNA methyltransferase inhibitor azacytidine is potentiated by esterification with the tFA elaidic acid (EA; tC18:1), suggesting that the latter may interact with chromatin [8].

To explore that issue, we asked whether EA modifies the DNA methylome and the transcriptome and whether such effects are distinct from the ones elicited by its *cis* isomer oleic acid (OA) in human THP-1 monocytes. We focused on EA and OA for their biological significance, as EA is one of the most abundant tFAs found in processed food and in circulation. Furthermore, OA has been attributed strikingly opposite, beneficial effects on human health compared to EA [9, 10], thus we assumed that differential epigenetic and transcriptional signatures between the two FAs were likely to be detectable. The rationale for using the THP-1 cell line as model is that it has been exploited to study the effects of lipoproteins and FAs on the DNA methylome ([3, 11] and our group’s unpublished data). In order to explore possible epigenetic long-term effects, we assessed whether EA shapes the DNA methylome in utero or during lactation in a mouse model. We discuss the results in the light of the current knowledge of FAs and disease risk.

## Results

### Effects of EA and OA on global DNA methylation

**Fig. 1:**
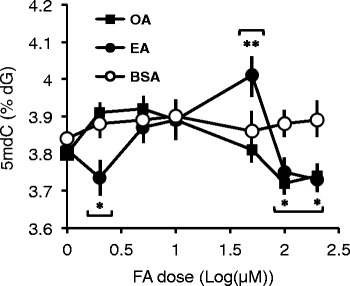
Effects of pure FAs on global DNA methylation in THP-1 monocytes following a 24-h stimulation. Data points represent averages and SD values of triplicate experiments. Asterisks above or below data points indicate the significance of the difference in comparison with the respective 1 μM dose (*, *p* < 0.05; **, *p* < 0.01; Mann–Whitney U test). Horizontal brackets indicate the EA or OA doses at which the response is significantly different (*p* < 0.05) from the response to the vehicle BSA

We first explored the effects of EA and OA on global DNA methylation, i.e. total 5mdC content calculated by an HPLC-based technique - in THP-1 monocytes. FAs were used in the 1–200 μM concentration range. These values are within the physiological range [12]. EA induced a biphasic effect on global DNA methylation, i.e. a hypermethylation in the 1–50 μM dose range corresponding to a 5.2 % increase in 5mdC levels, followed by a sharp hypomethylation up to the 200 μM dose (Fig. 1). On the other hand, OA exerted a similarly biphasic but weaker response peaking at 5 μM as previously reported (Silva-Martínez et al., in press). Furthermore, the response to OA did not significantly differ from the response induced by the carrier BSA up to the 50 μM dose. The maximal differential response between EA and OA was observed at 50 μM concentration.

### Whole genome expression analysis of EA- and OA-stimulated THP-1 monocytes

**Fig. 2:**
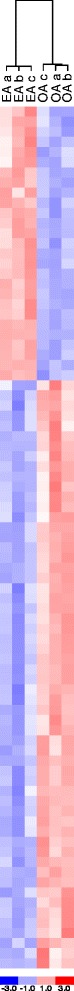
Clustering analysis of expression array data. Only the differentially expressed probes are shown. The red and blue color represent expression levels above and below the mean expression of a gene across all six arrays, respectively. a, b and c arbitrarily identify individual arrays according to the nomenclature used in the GEO database- deposited material

In order to understand the impact of the OA- and EA-induced changes in DNA methylation on gene expression, we performed a global genome expression analysis using the Affymetrix GeneChip® Human Genome U133 Plus 2.0 Array in THP-1 monocytes stimulated with 50 μM of either FA for 24 h. The rationale for using that FA concentration was that it corresponded to the maximal differential response between EA and OA in the global DNA methylation assay (see Fig. 1). The effects of 50 μM OA on global DNA methylation were not significantly different from the ones of BSA alone, therefore we only included EA- and OA-treated samples in the microarray analysis. Another reason for not including BSA-treated samples, is that because of the ubiquitous and continuous presence of FAs in the circulation, cultured cells not exposed to any FA do not have any physiological counterpart. A total of 137 probes corresponding to 100 annotated genes met the differential expression criteria of >2-fold absolute expression difference and *p* < 0.05 (t-test) (Additional file 1: Table S1). The majority (66 or 63.5 %) of the differentially expressed probes corresponding to annotated genes (104 probes, counting genes represented more than once) were down-regulated by EA. Expression datasets for the differentially expressed probes clearly clustered by FA treatment (Fig. 2). The functional enrichment analysis showed sharply distinct functions between genes up-regulated or down-regulated by EA (FDR < 0.05). The former were enriched in lipid biosynthesis activities, whereas the down-regulated genes were involved in cell cycle regulation (Table 1). A list of the EA-regulated genes belonging to those two functional categories and a summary of their function is shown in Additional file 1: Table S2. Other notable genes up-regulated by EA were pro-inflammatory factors (*IL20RB, IL21R, SLAMF7*), the cholesterol transporter *ABCA1*, chemokines (*CCL2, CCL4, CCL8, IL8*). Up-regulated genes were not significantly enriched for any transcription factor (TF) target category, but the down-regulated counterpart included targets for a number of TFs including interferon regulatory factor 2 (IRF2; FDR = 6.6 × 10^−7^). Among IRF2 targets were the inhibitors of inflammation myocyte enhancer factor 2C (*MEF2C*) and annexin A1, (*ANXA1*, also known as lipocortin 1), which was recently shown to decrease atherosclerosis in a mouse model [13–15]. The expression of a total of 11 genes was validated by reverse transcription-PCR (RT-PCR). Chemokines, inflammation-related and lipid metabolism-related genes including *ANXA1, CCL4* and *ADFP* were among the successfully validated differentially expressed probes (Additional file 2: Figure S1). Next, we asked whether any change in promoter DNA methylation was associated with differential gene expression between EA- and OA-stimulated cells. We profiled DNA methylation in the promoters of 7 of the genes for which expression was validated, by direct sequencing of bisulfite-modified genomic DNA. In addition to the CpG dinucleotide context, where methylated cytosines are predominantly found mammals, we analyzed by methylation of cytosines located in the 5′ position of trinucleotides with guanosine or any nucleotide at the 3′ position - i.e. CHG or CHH, where H represents non-G nucleotides (A, T or C). CHG and CHH methylation, although present at low frequency, has been detected in specific cell types and in atherosclerosis in humans, and may have regulatory functions [16, 17]. Our results showed only weak, non-significant changes between EA- and OA-stimulated cells in any of three contexts analyzed. DNA methylation profiling of *API5* and *PDK4* promoters are shown as examples in Additional file 2: Figure S2.

**Table 1:** Functional category enrichment among genes up-regulated or down-regulated by EA relative to OA in THP-1 monocytes (FDR < 0.05)

	GO term	FDR
Up-regulated	GO:0008610~lipid biosynthetic process	3.8 × 10^−4^
	GO:0006694~steroid biosynthetic process	0.006
	GO:0008202~steroid metabolic process	0.033
Down-regulated	GO:0000279~M phase	0.001
	GO:0022403~cell cycle phase	0.009
	GO:0007049~cell cycle	0.040

### Effects of EA on DNA methylation in the adipose tissue in mice

The above data indicated that EA exerts global DNA hypermethylation. To further corroborate those observations, we analyzed global DNA methylation in the progeny of dams supplemented with EA either during the whole pregnancy or lactation. The EA dose was 0.63 mg/day (0.7 μl) or the 30 g-body weight (BW) mouse equivalent of the tFA exposure of a 70 kg-human eating one generic cheeseburger every 3 days, which is slightly below recently calculated U.S.A. figures of 3 hamburgers/week (http://www.economist.com/node/154515?story_id=154515). EA did not have any significant effects on dam’s BW during pregnancy nor during lactation (*n* = 5 in each group). No adverse effects or macroscopic anatomical abnormalities were observed, with the exception of one case of fat necrosis in the mammary gland of one dam that had received EA during lactation.

**Fig. 3:**
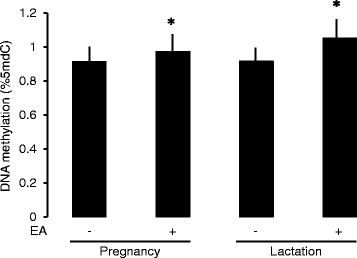
Effect of maternal EA supplementation on the progeny’s epididymal fat pad global DNA methylation. Data represent average and SD for 3 replicates, each representing 8–6 mice, in all experimental groups indicated. *, *p* < 0.05 (Mann–Whitney U test)

The progeny however, showed a significant decrease in BW gain between birth and weaning that was significant only in males. BW gain, calculated as percent change compared to BW at birth, decreased by 16.6 ± 1.7 % and 10.1 ± 0.9 % in the male progeny of dams supplemented with EA during pregnancy or lactation, respectively, compared to controls (*n* = 10 for either sex in either treatment; *p* < 0.05 in both cases, Scheffé’s *post hoc* test). Global DNA methylation was measured in the progeny’s abdominal adipose tissue at 3 months of age. The rationale for choosing the adipose tissue was that EA alters the adipocyte’s fat composition and physiology [18]. Data were obtained for 3 DNA pools in each of the 4 experimental groups, each pool corresponding to one litter. EA supplementation during either pregnancy or lactation caused a significant increase in DNA methylation (7.8 % and 14.4 %, respectively, *p* < 0.05, Mann–Whitney U test; MethylFlash assay) (Fig. 3).

## Discussion

We show that EA and OA exert distinct effects on global DNA methylation in human THP-1 monocytes. These global epigenome responses coincide with distinct whole transcriptome profiles, but are not matched by any difference in the promoter DNA methylation of any of the transcriptionally regulated genes tested. Our data suggest that EA may target mainly either gene-body or intergenic regulatory element DNA methylation. Accordingly, we recently showed that AA hypermethylates the gene-body of target genes and members of the *ALU* repeated family in the same THP-1 cell line used here (Silva-Martínez et al., in press). At any rate, we show that maternal EA supplementation during pregnancy or lactation induces changes in the progeny’s adipose tissue DNA methylation that are co-directional - i.e. hypermethylation in both cases - with the response to physiological doses of EA observed in THP-1 cells.

The transcriptional profile induced by EA in THP-1 cells is markedly adipogenic compared to OA, in accordance with published data obtained with EA or *trans* conjugated FAs [19, 20]. tFA-induced adipogenesis is accompanied by hepatic steatosis [19, 21–24], which may share underlying mechanisms with the fatty lesions observed here in one mammary gland of EA-treated dams. The marked adipogenic response to EA is not reflected in a BW gain. In fact, a sexually dimorphic, male-restricted decrease in BW was observed in the progeny of EA-treated dams. Our data are consistent with previous findings in models of prenatal and adult exposure [18, 25, 26], although contrasting results have been reported, showing no significant effect on BW [27, 28] or an increase in primates and rodents [29–31] and an association with obesity in humans [1]. Despite these inconsistencies, our data suggest that the exposure to EA in utero or during lactation slows growth in the birth-to-weaning period. Males are more vulnerable to adverse maternal factors than females, and various mechanisms have been proposed for this sex-specific response, including functional differences in the immune system (extensively reviewed in [32]). Our results do not allow to conclude whether these effects are due to differential immunity, increased catabolism or decreased appetite. In addition, based on the enrichment of cell cycle-related genes in the transcripts down-regulated by EA in THP-1 monocytes, it is conceivable that the reduced BW gain in males may be due at least in part to reduced cell proliferation. Interestingly, the observation that the effects of EA on BW tended to be more marked following supplementation during pregnancy compared to supplmentation through breastmilk, suggested a developmental stage-related differential vulnerability to EA that deserves further research. At any rate, the low postnatal growth is interesting from a clinical viewpoint, as it may contribute to understand the association between EA intake and increased cardiovascular risk [33–36].

Our results in THP-1 monocytes identify specific responses to EA compared to other FAs. We recently identified the genes involved in the G protein-coupled receptor signalling pathway as a major targets in the transcriptional regulation by AA and OA, whereas EA did not modulate that pathway, nor AA or OA modulated the lipid biosynthesis pathway (Silva-Martínez et al., in press). In the same study, we showed by comparative genomics that PA induces differential DNA methylation in genes belonging to that same pathway. These genomic responses single out EA as a biologically peculiar FA when compared to AA, OA and PA, in line with the extensive evidence linking specifically tFAs to human disease.

## Conclusions

We document that the tFA EA and its *cis* isomer OA exert distinct global DNA methylation and transcriptome profiles in cultured human cells. In addition, EA induces global changes in the adipose tissue DNA methylome that can be imposed by maternal exposure and are detectable postnatally for up to 3 months in a mouse model. Our data suggest that the deleterious effects of tFAs on human health may extend beyond any decrease in exposure due to legal control or voluntary abstention. However, further studies are required in order to identify the genes involved in EA-induced changes in the DNA methylome.

## Methods

### Cell culture and stimulation with FAs

THP-1 monocytes were cultured in RPMI-1640 medium (Gibco) supplemented with 2 mM L-Glutamine (Sigma), 10 % foetal calf serum (Gibco) and 1 % Penicillin/Streptomycin (Gibco). Cells were never allowed to grow above a 1–1.5 × 10^6^ cells/ml concentration. Pure FAs (Sigma) were conjugated with cell culture-grade FA-free BSA (fraction V, FA-free, Sigma no. 820022) to achieve a FA:BSA 6:1 ratio, essentially as described [37]. Typically, 5-6 × 10^6^ cells in 10 ml medium were stimulated with 100x BSA-FA mix in 2 % FCS. Exclusion of trypan blue was used as a criterion for viability.

### Animal experiments

Female C57BL/6 mice were assigned to each of the following 4 groups, *n* = 5 per group. 1) Supplemented with 0.7 μl EA dissolved in 5 μl commercial soybean oil vehicle (Nutrioli brand) orally once a day for the whole pregnancy period, i.e. from the detection of the vaginal plug to the day prior to delivery (20 days). 2) As in the previous group but supplemented with vehicle alone. 3) Supplemented with EA as in the first group but during the entire lactation, i.e. between birth and weaning (28 d *post partum*). 4) As in group 3 but with vehicle alone. Due to the small volume, a micropipette was carefully used for oral administrations and mice were extensively monitored for any sign of discomfort and to ensure complete swallowing. At the age of 3 months, a total of 20 progeny mice - 10 for each sex - were randomly chosen from 3 of the most numerous litters, 8–6 siblings/litter, and dissected to obtain the epididymal fat pad for the downstream analyses. The choice of the three most numerous litter aimed at minimizing a possible effect of litter size. The post-exposure time at dissection was therefore 3 months and ~2 months for the pregnancy and lactation supplementation groups, respectively. The procedure was approved by the Ethical Committee of the Department of Medical Sciences of the University of Guanajuato.

### Gene expression

Whole-genome expression data were obtained with the Affymetrix GeneChip Human Genome U133 Plus 2.0. Arrays were hybridized with labelled total RNA extracted by using the RNeasy system (Qiagen), scanned with an Affymetrix GeneChip Scanner 3000 according to standard protocols at the microarray facility, Rigshospitalet, Copenhagen, Denmark. RNA integrity was checked by agarose electrophoresis at the source laboratory and again at the microarray facility. The dChip software (build April 15, 2005) was used for normalization and modelling using the PM-only model. Clustering was performed with dChip. Expression microarray data are publicly available in the GEO database with accession number GSE74874. Validation was performed by RT-PCR using primers designed with the qPrimerDepot tool (primerdepot.nci.nih.gov). The primers listed in Additional file 1: Table S3.

### DNA methylation analysis

DNA was extracted from cultured cells by standard methods (DNeasy system, Qiagen). Global DNA methylation was measured in THP-1 cells by a HPLC-based method yielding 5-methyl-2′-deoxycytidine (5mdC) values normalized by 2′-deoxyguanosine (dG) [38]. Global DNA methylation in mice was measured by the MethylFlash system (Epigentek) according to the manufacturer’s instructions, using DNA extracted from one randomly chosen fragment of epididymal fat pad in each animal. The assay output is the percent 5-methyl-2′-deoxycytidine (5mdC) of PicoGreen-quantified input genomic DNA (wt/wt). The difference in the DNA methylation levels obtained with the two assays is therefore explained by the distinct normalization methods. Gene promoter DNA methylation profiling was carried out by sequencing at least 10 clones per FA treatment obtained by PCR amplification of bisulfite-treated genomic DNA. Primers were designed using the BiSearch tool (http://bisearch.enzim.hu) [39]. Primer sequences are listed in Additional file 1: Table S3.

### Statistics

Comparisons were carried out by the Mann–Whitney U test. The only exception was the expression array data, where we used the t-test. The Kruskal-Wallis test followed by the Scheffé’s ANOVA *post hoc* test were applied to compare control and treated male and female mouse data. All tests were performed with the STATISTICA (StatSoft) software.

## Additional files

Additional file 1: Table S1. Differentially expressed probes between EA and OA stimulated cells. **Table S2.** Genes differentially expressed in EA and OA stimulated cells for the significantly enriched functional gene categories listed in main text Table 1. **Table S3.** Primers used in RT-PCR and bisulfite-modified DNA sequencing. (PDF 134 kb) Additional file 2: Figure S1. Validation of selected expression array data for FA-stimulated THP-1 cells. RT-PCR results are shown for the indicated genes, performed in triplicate. **Figure S2.** Context-specific DNA methylation profiling of *API5* and *PDK4* promoters. Contexts (CG, CHG and CHH, where H indicates a non-G nucleotide *-* i.e. A, C or T. Open bars: OA. Solid bars: EA. (PPTX 1043 kb)
