# Supplementary material for: The trans fatty acid elaidate affects the global DNA methylation profile of cultured cells and in vivo
Source: Lipids Health Dis. 2016 Apr 12;15:75. doi: 10.1186/s12944-016-0243-2 (PMC4828757; doi:10.1186/s12944-016-0243-2)
Supplement: Additional file 1: Table S1. — Differentially expressed probes between EA and OA stimulated cells. Table S2. Genes differentially expressed in EA and OA stimulated cells for the significantly enriched functional gene categories listed in main text Table 1. Table S3. Primers used in RT-PCR and bisulfite-modified DNA sequencing. (PDF 134 kb) [file 12944_2016_243_MOESM1_ESM.pdf]

Additional file 1: Table S1 - Differentially expressed probes between EA- and OA-stimulated cells.

Probe set	Gene	P value	Accession	EntrezGene	Description	EA/OA fold change
225207_at	PDK4	0.033	AV707102	5166	Hs.8364.0	5.32
235805_at	ACSS2	0.000	AA706922		Hs.189998.0	4.59
1557252_at	CDNA FLJ36213 fis, clone THYMU2000671	0.013	AK093532		Hs2.114526.1	3.45
211019_s_at	LSS	0.011	D63807	4047	g1019365	3.2
205822_s_at	HMGCS1	0.017	NM_002130	3157	g4504428	3.07
1569496_s_at	SPON2	0.001	BC030552	10417	Hs2.382136.1	2.98
204580_at	MMP12	0.043	NM_002426	4321	g4505206	2.9
221658_s_at	IL21R	0.000	AF269133	50615	g10801190	2.87
220675_s_at	PNPLA3	0.001	NM_025225	80339	g13376829	2.85
211162_x_at	SCD	0.029	AF116616	6319	g7959734	2.74
204103_at	CCL4	0.014	NM_002984	6351	g4506844	2.72
219971_at	IL21R	0.000	NM_021798	50615	g11141868	2.7
209122_at	ADFP	0.027	BC005127	123	g13477306	2.63
212272_at	LPIN1	0.021	AA813260	23175	Hs.81412.0	2.59
210173_at	PTPRJ	0.043	D37781	5795	g633072	2.58
217078_s_at	CD300A	0.001	AJ010102	11314	Hs.9688.1	2.57
212143_s_at	IGFBP3	0.005	BF340228	3486	Hs.77326.1	2.53
222486_s_at	ADAMTS1	0.005	AF060152	9510	g5725505	2.5
214038_at	CCL8	0.005	AI984980	6355	Hs.271387.0	2.47
210701_at	CFDP1	0.027	D85939	10428	g2114175	2.47
203027_s_at	MVD	0.009	AI189359	4597	Hs.3828.0	2.45
239439_at	AFF4	0.045	BF963382	27125	Hs.88808.0	2.45
203504_s_at	ABCA1	0.006	NM_005502	19	g5915657	2.37
234312_s_at	ACSS2	0.000	AK000162	55902	Hs.14779.1	2.36
205069_s_at	ARHGAP26	0.044	NM_015071	23092	g7662207	2.32
235779_at	LOC284408	0.041	AW467077	284408	Hs.103362.0	2.31
1556314_a_at	(clone 1NIB-4) normalized cDNA library sequence	0.037	BQ002274		Hs2.224794.1	2.3
211018_at	LSS	0.002	D63807	4047	g1019365	2.28
211708_s_at	SCD	0.032	BC005807	6319	g13543283	2.27
238449_at	LOC595101	0.035	BG534511	595101	Hs.331640.0	2.26
1557418_at	ACSL4	0.030	W95007	2182	Hs2.269559.1	2.25
241716_at	HSPD1	0.018	BF965447	3329	Hs.160227.0	2.25
1552553_a_at	NLRC4	0.010	NM_021209	58484	Hs2.23248.1	2.22
211506_s_at	IL8	0.013	AF043337	3576	g12641914	2.2
201169_s_at	BHLHB2	0.032	BG326045	8553	Hs.171825.0	2.2
1569392_at	GPSN2	0.025	BC034484	9524	Hs2.98153.1	2.2
237753_at	IL21R	0.001	AW504569		Hs.126232.0	2.19
209875_s_at	SPP1	0.016	M83248	6696	g189150	2.18
1554152_a_at	OGDH	0.032	BC009580	4967	Hs2.168669.2	2.18
232861_at	PDP2	0.042	AB037769	57546	Hs.232696.0	2.17
233030_at	PNPLA3	0.002	AK025665	80339	Hs.266738.1	2.14
228575_at	IL20RB	0.042	AL578102	53833	Hs.61232.0	2.14
239358_at	FDFT1	0.034	BE645256		Hs.94151.0	2.12
225742_at	MDM4	0.051	W93501	4194	Hs.31433.0	2.08
215791_at	Chromosome 21q22.1 anonymous mRNA sequence	0.028	AF003738		Hs.268526.0	2.08
202218_s_at	FADS2	0.001	NM_004265	9415	g4758333	2.07
216598_s_at	CCL2	0.012	S69738	6347	Hs.303649.1	2.07
221211_s_at	C21orf7	0.001	NM_020152	56911	g9910145	2.07
1555996_s_at	EIF4A2	0.044	AI332397	1974	Hs2.410186.1	2.07
222838_at	SLAMF7	0.011	AL121985	57823	Hs.132906.0	2.05
222162_s_at	ADAMTS1	0.009	AK023795	9510	Hs.8230.1	2.04
1553137_s_at	KLF11	0.028	AF028008	8462	Hs2.408442.1	2.03
1554948_at	Hs2.375076.1	0.028	BC034024		Hs2.375076.1	2.02
222209_s_at	TMEM135	0.001	AK000684	65084	Hs.183887.1	2.01
208860_s_at	ATRX /// LOC728849	0.001	U09820	546 /// 728849	g606832	-2.01
204840_s_at	EEA1	0.000	AI916242	8411	Hs.2864.0	-2.01
232297_at	CDNA FLJ11313 fis, clone PLACE1010106, highly similar to Homo sapiens mRNA; cDNA DKFZp586M1418	0.003	AL049385		Hs.272251.0	-2.02
227987_at	VPS13A	0.000	AW629014	23230	Hs.65919.0	-2.02
221078_s_at	CCDC88A	0.022	NM_018084	55704	g8922402	-2.02
1552309_a_at	NEXN	0.002	NM_144573	91624	Hs2.216381.1	-2.02

225045_at	CCDC88A	0.002	AB033038	55704	Hs.20887.1	-2.03
201731_s_at	TPR	0.001	NM_003292	7175	g4507658	-2.04
210962_s_at	AKAP9	0.004	AB019691	10142	g5051742	-2.04
215220_s_at	TPR	0.034	AK023111	7175	Hs.306642.0	-2.04
214700_x_at	RIF1	0.008	AK000323	55183	Hs.225841.1	-2.05
236623_at	C1orf203	0.027	AI367432	84852	Hs.133509.0	-2.05
1569652_at	MLLT3	0.030	BC030550	4300	Hs2.382134.1	-2.05
231872_at	LRRCC1	0.006	T52285	85444	Hs.193115.0	-2.06
213239_at	C13orf24	0.001	NM_006346	10464	Hs.43913.0	-2.07
204841_s_at	EEA1	0.031	NM_003566	8411	g4503468	-2.07
242352_at	NIPBL	0.008	AW272262	25836	Hs.250468.0	-2.08
230165_at	SGOL2	0.005	N31731	151246	Hs.44269.0	-2.1
236620_at	RIF1	0.003	AU150841	55183	Hs.254020.0	-2.1
1562484_at	FLJ35848	0.000	BC041481	284071	Hs2.309103.3	-2.11
207828_s_at	CENPF	0.002	NM_005196	1063	g4885132	-2.12
1564776_at	LENG10	0.001	AF211977	94057	Hs2.404144.1	-2.12
1552680_a_at	CASC5	0.002	NM_020380	57082	Hs2.283099.2	-2.12
222313_at	CNOT3	0.002	AW972359		Hs.293334.0	-2.13
1562230_at	Full length insert cDNA clone YI60C11	0.007	AF147390		Hs2.384152.1	-2.14
236128_at	ZNF91	0.009	AI672373	7644	Hs.183950.0	-2.15
230020_at	LSM8	0.002	AI536000		Hs.122460.0	-2.15
227934_at	Hs.24252.0	0.003	AI769559		Hs.24252.0	-2.16
238443_at	TFAM	0.038	BE546873	7019	Hs.198113.0	-2.16
218859_s_at	ESF1	0.004	NM_016649	51575	g7705402	-2.18
243509_at	BTG1	0.031	AI475680		Hs.105676.0	-2.18
204291_at	ZNF518	0.000	NM_014803	9849	g7662059	-2.2
205046_at	CENPE	0.013	NM_001813	1062	g4502780	-2.21
238759_at	CCDC88A	0.032	AI681810	55704	Hs.244532.0	-2.21
226604_at	TMTC3	0.002	AA418403	160418	Hs.29643.0	-2.22
241853_at	Transcribed locus	0.001	AA760878		Hs.38485.0	-2.22
1553725_s_at	ZNF644	0.031	NM_016620	84146	Hs2.334470.1	-2.23
201057_s_at	GOLGB1	0.002	NM_004487	2804	g4758453	-2.24
228709_at	TPR	0.001	BF109993	7175	Hs.218791.1	-2.24
213957_s_at	CEP350	0.025	AA635523	9857	Hs.92200.1	-2.25
209778_at	TRIP11	0.011	AF007217	9321	g2253416	-2.26
205235_s_at	MPHOSPH1	0.011	NM_016195	9585	g7705347	-2.27
209172_s_at	CENPF	0.010	U30872	1063	g1000093	-2.28
235427_at	CASP8	0.002	AA418074		Hs.110286.0	-2.28
1558293_at	KIAA1107	0.003	BC037317	23285	Hs2.398145.1	-2.28
201567_s_at	GOLGA4	0.003	NM_002078	2803	g6715599	-2.31
208859_s_at	ATRX /// LOC728849	0.005	AI650257	546 /// 728849	Hs.96264.0	-2.31
235009_at	FAM44A	0.008	AI049791	259282	Hs.205442.0	-2.31
230064_at	Transcribed locus	0.005	AW296421		Hs.121035.0	-2.31
233219_at	CDNA FLJ13365 fis, clone PLACE1000332	0.000	AK023427		Hs.306646.0	-2.33
215767_at	ZNF804A	0.003	AF052145	91752	Hs.159528.0	-2.34
229066_at	Transcribed locus	0.001	AA694209		Hs.67197.0	-2.34
1556744_a_at	ZNF654	0.000	AI732587	55279	Hs2.118758.1	-2.35
219918_s_at	ASPM	0.002	NM_018123	259266	g8922484	-2.36
202319_at	SENP6	0.003	NM_015571	26054	g7662311	-2.37
241820_at	RIF1	0.023	BF666241	55183	Hs.254020.1	-2.37
239236_at	PHF10	0.008	BE465277		Hs.280730.0	-2.37
213222_at	PLCB1	0.002	AL049593	23236	Hs.41143.0	-2.38
219469_at	DYNC2H1	0.001	NM_024606	79659	g13375812	-2.39
207117_at	ZNF117	0.023	NM_015852	51351	g7705388	-2.42
238853_at	RAB3IP	0.010	AI436813	117177	Hs.283645.0	-2.42
201730_s_at	TPR	0.005	BF110993	7175	Hs.169750.0	-2.43
236395_at	MEF2C	0.002	AI523245		Hs.127638.0	-2.43
231855_at	KIAA1524	0.006	AB040957	57650	Hs.151343.0	-2.43
236160_at	hypothetical LOC341378; thyroid hormone receptor interactor 11	0.006	AA765470		Hs.122826.0	-2.43
214727_at	BRCA2	0.003	X95152	675	Hs.34012.1	-2.44
1558250_s_at	CDNA FLJ40891 fis, clone UTERU2001110	0.000	AI538880		Hs2.399730.1	-2.46
219644_at	CCDC41	0.001	NM_016122	51134	g7705838	-2.46
205250_s_at	CEP290	0.006	NM_014684	80184	g7662079	-2.5

206931_at	ZNF141	0.004	NM_003441	7700	g4507992	-2.52
212289_at	ANKRD12	0.005	AB020681	23253	Hs.27973.0	-2.53
205705_at	ANKRD26	0.008	AL137351	22852	Hs.129218.0	-2.55
1555352_at	FOXP2	0.012	AF467257	93986	Hs2Affx.1.231	-2.56
208393_s_at	RAD50	0.001	NM_005732	10111	g5032016	-2.61
219754_at	RBM41	0.006	NM_018301	55285	g8922825	-2.64
1557227_s_at	TPR	0.017	AW235355	7175	Hs2.433856.1	-2.65
216563_at	ANKRD12	0.023	X80821	23253	Hs.302177.0	-2.65
233078_at	API5	0.003	AK021649	8539	Hs.122518.0	-2.66
205706_s_at	ANKRD26	0.005	NM_014915	22852	g7662473	-2.8
232760_at	TEX15	0.007	AL133653	56154	Hs.272315.0	-2.97
212286_at	ANKRD12	0.016	AW572909	23253	Hs.27973.0	-3.2
220095_at	C9orf39	0.010	NM_017738	54875	g8923250	-3.56
233011_at	ANXA1	0.022	AU155094	301	Hs.287558.0	-3.73

Additional file 1: Table S2 - Genes differentially expressed in EA- and OA-stimulated cells for the significantly enriched functional gene categories listed in main text Table 1.

Gene		Function
**Up-regulated by EA**		
3-hydroxy-3-methylglutaryl-Coenzyme A synthase 1 (soluble)	HMGCS1	Participates in cholesterol synthesis
acyl-CoA synthetase short-chain family member 2	ACSS2	Produces acetyl-CoA from acetate for lipid synthesis
farnesyl-diphosphate farnesyltransferase 1	FDFT1	First specific enzyme in cholesterol biosynthesis, forms squalene
fatty acid desaturase 2	FADS2	Regulates FA desaturation
glycoprotein, synaptic 2	TECR	Participates in FA elongation
hydroxysteroid (17-beta) dehydrogenase 12	HSD17B12	Participates in FA elongation and in the conversion of estrone to estradiol
lanosterol synthase (2,3-oxidosqualene-lanosterol cyclase)	LSS	Participates in the sysnthesis of cholesterol, steroid hormones, and vitamin D
mevalonate (diphospho) decarboxylase	MVD	catalyzes the conversion of mevalonate pyrophosphate into isopentenyl pyrophosphate in cholesterol biosynthesis
patatin-like phospholipase domain containing 3	PNPLA3	Triacylglycerol lipase (adipocyte)
stearoyl-CoA desaturase (delta-9-desaturase)	SCD	Participates in FA biosynthesis
ATP binding cassette subfamily A member 1	ABCA1	Participates in cellular lipid renoval by promoting cholesterol efflux
**Down-regulated by EA**		
shugoshin-like 2 (S. pombe)	SGOL2	Participates in chromosome segregation in meiosis
leucine rich repeat and coiled-coil centrosomal protein 1	LRRCC1	Associates with the centrosome during mitosis
Nipped-B homolog (Drosophila)	NIPBL	Participates in sister chromatid cohesion, chromosome condensation and DNA repair. Linked to the Cornelia de Lange syndrome
testis expressed 15	TEX15	Involved in meiosis
centromere protein F	CENPF	May play a role in chromosome segregation during mitotis
centromere protein E	CENPE	Participates in chromosome segregation in mitosis
abnormal spindle microtubule assembly	ASPM	Essential for normal mitotic spindle function
kinesin family member 20B	KIF20B (MPHOSPH1)	Required for cytokinesis
RAD50 double strand break repair protein	RAD50	Pleiotropic, participates in cell cycle checkpoint activation
replication timing regulatory factor 1	RIF1	Regulates DNA replication and repair
cancer susceptibility candidate 5	CASC5	Participates in chromosome segregation in mitosis

Additional file 1: Table S3 - Primers used in RT-PCR and bisulfite-modified DNA sequencing.

RT-PCR	
Gene	Primer
ADFP	TCCTCAATCCTGTCTAGCCC
	GAGATGGCAGAGAACGGTGT
ANKRD12	AACCCAGATTTGGGCATTTT
	CGAAGACAACGACAGCGAC
ANKRD26	ATGCCTGGCTTTGTTGGTT
	TTGTGGTTGAAAGCCTTCCT
ANXA1	CACCTTTGGATGACTTCACAGT
	TCTTTGCAAGAAGGTAGAGATAAAG
API5	GAGCTGCTAATCGCTTTTCC
	CTCACCATGCCGACAGTAGA
CCL4	GATTCACTGGGATCAGCACA
	CTTTTCTTACACCGCGAGGA
GAPDH	AATGAAGGGGTCATTGATGG
	AAGGTGAAGGTCGGAGTCAA
HMGCS1	CTTCAGGTTCTGCTGCTGTG
	CAGAAGAACTTACGCTCGGC
IGFBP3	CTTGGGATCAGACACCCG
	GTCAACGCTAGTGCCGTCAG
IL21R	AATCGGTGTAGCAGACGAGG
	AGCACACGGAATGGATTTCT
PDK4	CACGATGTGAATTGGTTGGT
	TGATGTGGTAGCAGTGGTCC
SPON2	TAGCGAGTCCTGCTCTTGGT
	CCAGCAGGGACAATGAGATT
Bisulfite-modified DNA sequencing	
Gene	
ADFP	TGTTGGTAGGAAAGAAGTTTTAA
	CCAATTAAAATAAAAAACTCCCC
ANKRD12	GTGTAAAAATAATAGGTGAA
	CAAACACTATCCRACAAAAAAAAA
ANKRD26	TTGGGTGGTTTTTATAAAAG
	CATAAAATATCTCTAAAACCC
ANXA1	CACCTTTGGATGACTTCACAGT
	TCTTTGCAAGAAGGTAGAGATAAAG
API5	GGGTTTTTTAATAGTATAGG
	CTTAAACTTAACTACAAACTCT
PDK4	AAATGTTTTATTTTTYGGGGTAT
	AACTCCTCCTATTTAAAACT
SPON2	GGGATTAGATTTTTAAAGTT
	AAACTAAAACCTCATTAACC

Primer sequences are 5'-3'. R = A or G. Y = C or T.
