# Supplementary material for: The trans fatty acid elaidate affects the global DNA methylation profile of cultured cells and in vivo
Source: Lipids Health Dis. 2016 Apr 12;15:75. doi: 10.1186/s12944-016-0243-2 (PMC4828757; doi:10.1186/s12944-016-0243-2)
Supplement: Additional file 2: Figure S1. — Validation of selected expression array data for FA-stimulated THP-1 cells. RT-PCR results are shown for the indicated genes, performed in triplicate. Figure S2. Context-specific DNA methylation profiling of API5 and PDK4 promoters. Contexts (CG, CHG and CHH, where H indicates a non-G nucleotide - i.e. A, C or T. Open bars: OA. Solid bars: EA. (PPTX 1043 kb) [file 12944_2016_243_MOESM2_ESM.pptx]

## Slide 1
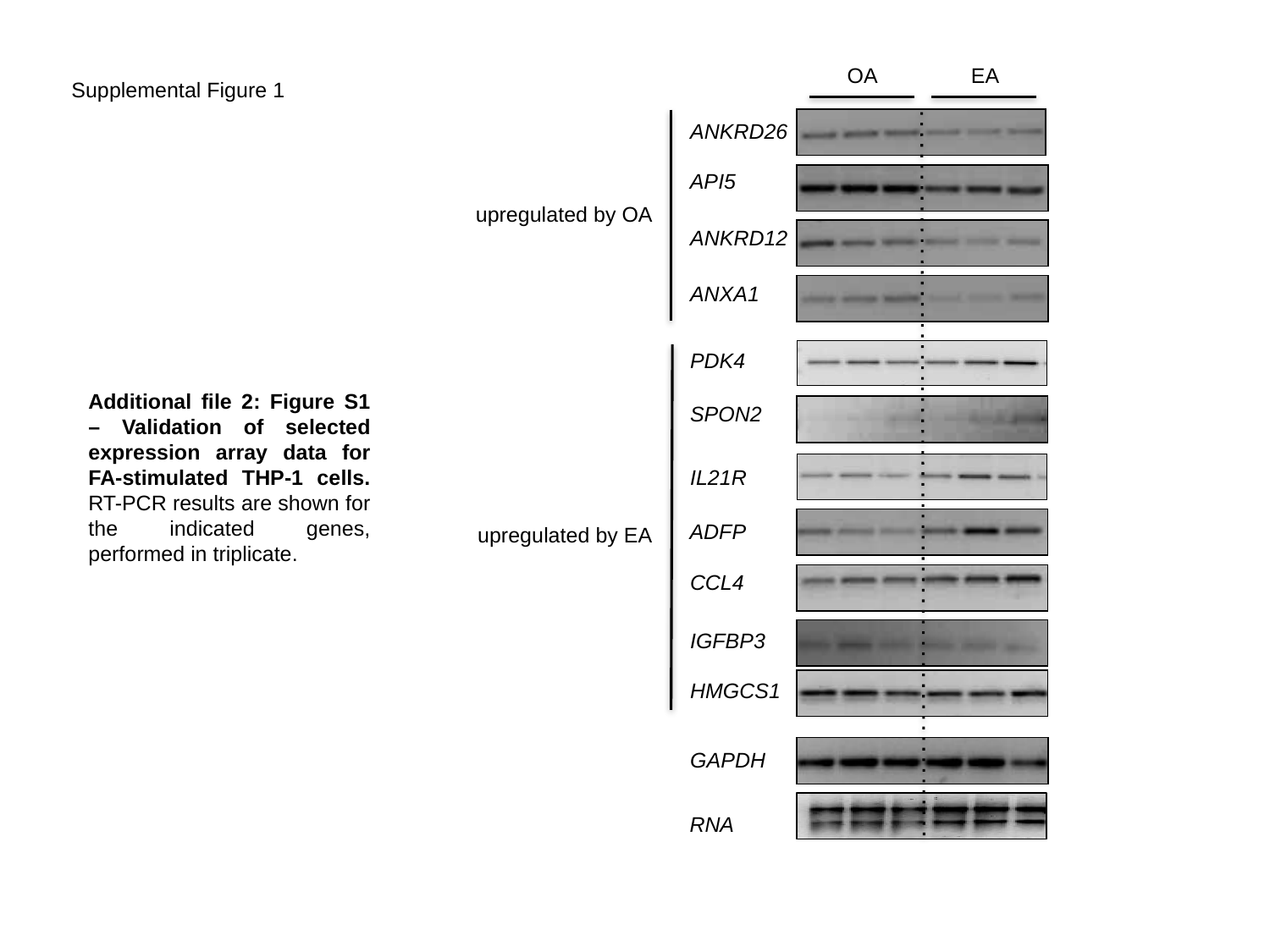

OA EA
Supplemental Figure 1
ANKRD26
API5
upregulated by OA
ANKRD12
ANXA1
PDK4
Additional file 2: Figure S1 – Validation of selected expression array data for FA-stimulated THP-1 cells. RT-PCR results are shown for the indicated genes, performed in triplicate.
SPON2
IL21R
ADFP
upregulated by EA
CCL4
IGFBP3
HMGCS1
GAPDH
RNA

## Slide 2
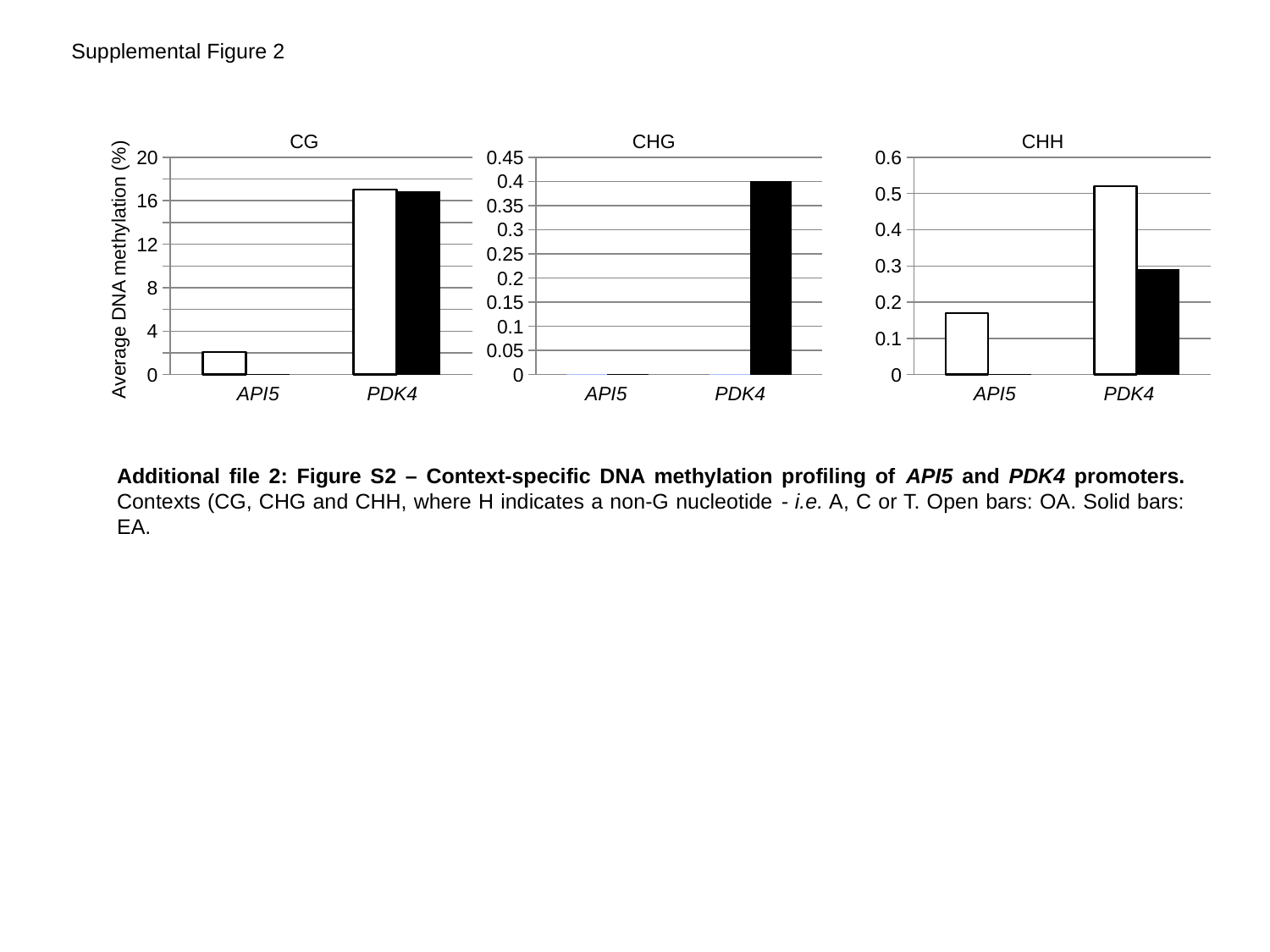

Supplemental Figure 2
CG
CHG
CHH
### Chart
| Category | OA CG | EA CG |
|---|---|---|
| API-5 | 2.08 | 0.0 |
| PDK4 | 17.02 | 16.8 |
### Chart
| Category | OA CNG | EA CNG |
|---|---|---|
| API-5 | 0.0 | 0.0 |
| PDK4 | 0.0 | 0.4 |
### Chart
| Category | OA CNN | EA CNN |
|---|---|---|
| API-5 | 0.17 | 0.0 |
| PDK4 | 0.52 | 0.29000000000000004 |Average DNA methylation (%)
API5 PDK4
API5 PDK4
API5 PDK4
Additional file 2: Figure S2 – Context-specific DNA methylation profiling of API5 and PDK4 promoters. Contexts (CG, CHG and CHH, where H indicates a non-G nucleotide - i.e. A, C or T. Open bars: OA. Solid bars: EA.
